# *Paratullbergia* Womersley in China: the description of a new species and a key to the genus (Collembola, Tullbergiidae)

**DOI:** 10.3897/zookeys.534.6036

**Published:** 2015-11-11

**Authors:** Yun Bu, Yan Gao

**Affiliations:** 1 Natural History Research Center, Shanghai Natural History Museum, Shanghai Science & Technology Museum, Shanghai, 200041, China; 2 Shanghai Hengjie Chemical Co. Ltd., Shanghai, 201599, China

**Keywords:** Identification key, taxonomy, pseudocelli, chaetotaxy

## Abstract

The genus *Paratullbergia* Womersley, 1930 is recorded for the first time from China. *Paratullbergia changfengensis* **sp. n.** from Shanghai is described and illustrated. It is characterized by the presence of 1+1 pseudocelli on thoracic segment I, with two pairs of pseudocelli on each of thoracic segments II and III, presence of seta px on abdominal segment IV, seta a2 and p4 on abdominal segment V as microsetae, and less differentiated sensory seta p3 on abdominal segment V. Both sexes present. The new species can be easily distinguished from its congeners by the presence of pseudocelli on thoracic segment I. An updated key to the world species of the genus *Paratullbergia* is provided.

## Introduction

The family Tullbergiidae Bagnall, 1947 contains a group of tiny euedaphic collembolans with approximately 200 species reported in the world (Bellinger et al. 2015); however, the Chinese Tullbergiidae are poorly known, with only four species recorded to date (Rusek 1967; Tamura and Zhao 1996; Gao 2007; Bu et al. 2013). During the study of the collembolan collections from Changfeng Park of Shanghai, one new species belonging to the genus *Paratullbergia* Womersley, 1930 was identified and is described in the present paper.

The genus *Paratullbergia* contains eight species occurring in Holarctic, India, South Africa, and Australia (Prabhoo 1971; Rusek 1991; Dunger and Schlitt 2011; Bellinger et al. 2015). The habitus is similar to that of the genus *Mesaphorura* Rusek, 1973, with some species being robust, and having an integument with coarse granulation, antennal segment IV with 2 subapical sensory rods, antennal segment III with two large sensory clubs, bent towards one another and two small sensory rods, postantennal organ with 35-68 vesicles in 2-4 rows, and pseudocelli of type IV and type I. Asp stronger, longer than in *Mesaphorura*.

## Materials and methods

Specimens were collected by Berlese-Tullgren funnels and preserved in 80% ethanol. The material was mounted on slides in Hoyer’s solution and dried in an oven at 45 °C for identification. Drawings were done with the aid of a phase contrast microscope. The type specimens are deposited in Shanghai Natural History Museum (SNHM), Shanghai, China.

Abbreviations used in the descriptions:

Th. thoracic segment;

Abd. abdominal segment;

Ant. antennal segment;

s sensillum;

PAO postantennal organ;

a anterior setae;

m medial setae;

p posterior setae;

pl pleural setae;

pso pseudocelli.

## Results

### Taxonomy

#### Paratullbergia changfengensis sp. n.

Taxon classificationAnimaliaCollembolaTullbergiidae

http://zoobank.org/2E6BA83A-245D-4B94-89DA-99257167E83C

Figs 1–6 , Table 1

##### Material examined.

Holotype, male (No. Changfeng2-2) (SNHM), China, Shanghai, extracted from soil samples of broad-leaved forest of Changfeng Park, 31°13'N 121°23'E, 15-III-2015, coll. Y. Bu & Y. Gao. Paratypes, 2 females (Nos. Changfeng2-1, Changfeng3-Changfeng7) (SNHM), data same as holotype. Other materials: 2 juveniles and 1 male subadult (Nos. Changfeng1, Changfeng8, Changfeng9), data same as holotype.

##### Description.

**Figures 1–6.**
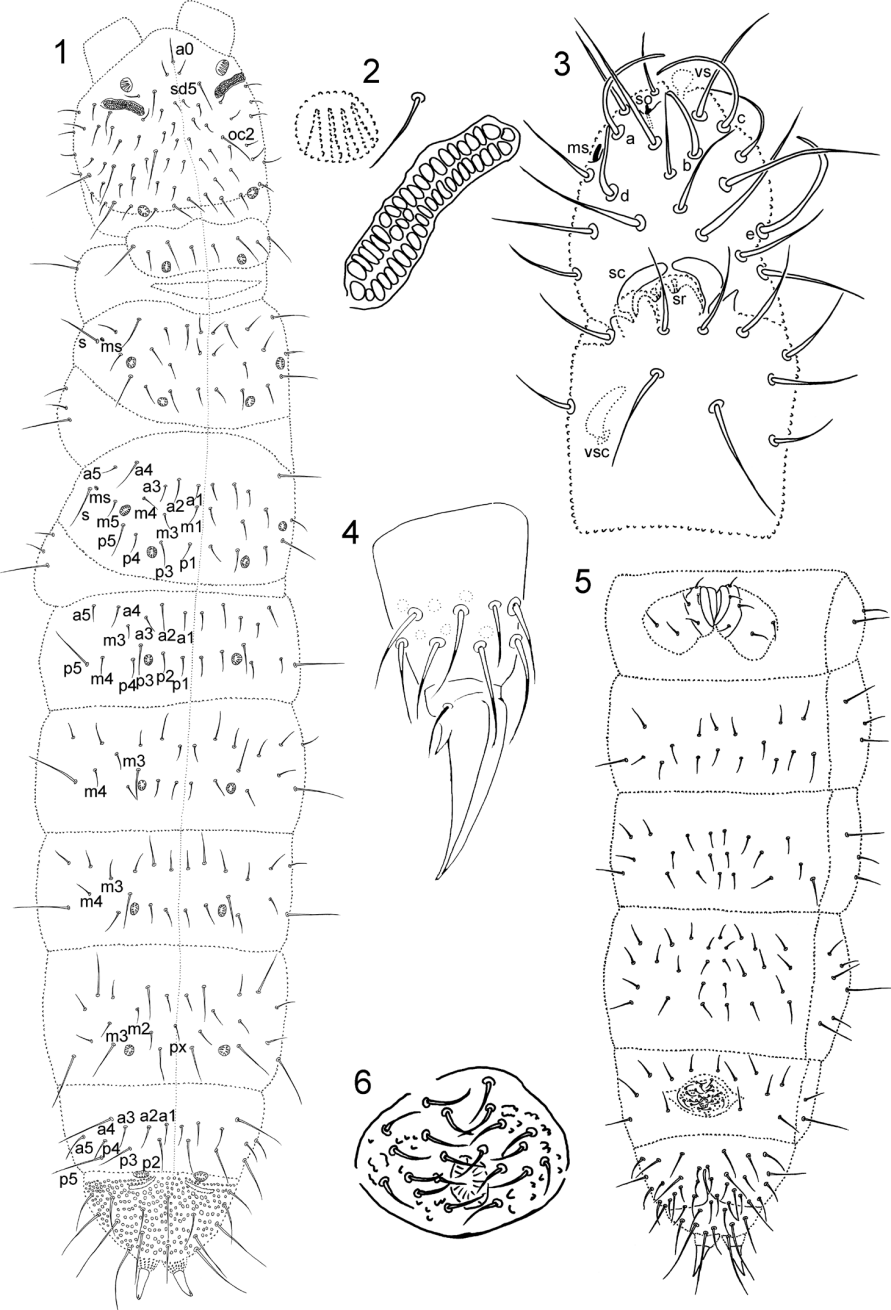
*Paratullbergia changfengensis* sp. n. (holotype). **1** Habitus, dorsal view, s–sensillum, ms–microsensillum
**2** Postantennal organ and pseudocelli **3** Ant. III and IV, a, b, c, d, e–large sensilla, ms–microsensillum, so–subapical organite, vs–apical vesicles, sc–sensory clubs, sr–sensory rods, vsc–ventral sensory club
**4** Tibiotarsus III and claw **5** Abdomen, ventral view **6** Male genital plate.

Adult body 0.85 mm long in average (0.7–1.0 mm, n = 7). Both females and males were present. Setae well differentiated into micro- and macrosetae (Fig. 1). Granulations coarse, formed by secondary granules, 2.5–4.0 µm in diameter. Pseudocellar formula: 11/122/11111, 5–8 µm in diameter; on antenna base composed by four ridges from one side only (type IV) (Fig. 2), others star-like (type I); on Th. I between seta m2 and m3, and close to hind margin; on Th. II and III between setae m4/m5 and p3/p4, and close to m5 and p3 respectively; on Abd. I–III posterior to seta p3; on Abd. IV parallel to seta p3; on Abd. V on the border of Abd. VI (Fig. 1).

Head seta a0 present (20–22 µm), c1 absent, oc2 as macroseta (25–30 µm), and sd5 as mesoseta (20–25 µm) (Fig. 1). Postantennal organ 25–30 µm long and 6–7 µm wide, composed of 32–47 elliptical vesicles arranged in two rows, and latero-externally widened with three rounded vesicles inserted (Fig. 2). Labrum with 4/5/4 setae. Labium with five papillae, six apical guard setae, six proximal setae, four basomedian setae, and five basolateral setae.

Antenna (100–135 µm) shorter than head (130–150 µm). Ant. segment IV (Fig. 3) with five slightly thickened sensilla a–e, without basal heel, sensilla a, c, e long and curved toward inside, b and d short. Small microsensillum, subapical organite and one large apical vesicles present. Antennal organ III (Fig. 3) consists of two small sensory rods concealed behind two papilla and two thick sensory clubs bent toward each other, with four guard setae.

Legs without clavate tibiotarsal hairs (Fig. 4). Coxa, trochanter, femur and tibiotarsus with 3/7/7; 6/6/5; 10/10/10; 15/15/14 setae on Leg I, II and III, respectively. Anal lobes with seta 12’ and l3’ (Fig. 5). Claw 25 µm long, with short empodial appendage. Anal spines 30–32 µm long.

Adult chaetotaxy given in Fig. 1, Fig. 5 and Table 1. Microsensilla present on Th. II-III, and lateral sensory setae s 37–39 µm long (Fig. 1). Thorax with 0, 2, 2 ventral setae. Abd. I–III each with 2+2 axial setae dorsally, setae m3 and m4 present. Abd. IV with seta px, setae m2 and m3 present. Abd. segment V with sensory seta p3 slightly differentiated, 22–29 µm long; seta a2 (15–16 µm) and p4 (19–21 µm) as microsetae (Fig. 1). Crescentic ridges on Abd. VI present.

Number of ventral setae on Abd. II, III and IV variable, with 17–20, 19–23, and 22–26 setae respectively (Fig. 5). Ventral tube with 4+4 apical setae and 2+2 basal setae (Fig. 5). Male genital plate with 18-21 setae (Fig. 6).

**Table 1.** Adult Chaetotaxy of *Paratullbergia changfengensis* sp. n.

		Thorax			Abdomen				
Segments		I	II	III	I	II	III	IV	V
Dorsal	a	-	10	10	10	10	10	10	10^4^
	m	8	8	8	4^1^	4^1^	4^1^	4^2^	-
	p	-	8	8	10	10	10	11^3^	8^5^
	pl	2	3	3	2	3	3	6	2
Ventral		0	2	2	12	17–20	19–23	22–26	10+(18–21)+4^6^

1 seta m3 and m4 present

2 seta m2 and m3 present

3 seta px present

4 seta a2 as microseta

5 sensory seta p3 slightly differentiated, seta p4 as microseta

6 male genital plate with 18–21 setae

##### Etymology.

The species is named after the Changfeng Park where the type specimens were collected.

##### Distribution.

Known only from the type locality. Considering that all specimens were only found in Changfeng Park, and that no other *Paratullbergia* has ever been recorded from China, this species has been probably introduced from an other place together with plants and soil.

##### Diagnosis.

*Paratullbergia changfengensis* sp. n. is characterized by the presence of pseudocelli on thoracic segment I, with two pairs of pseudocelli on each thoracic segment II and III, the presence of seta px on abdominal segment IV, setae a2 and p4 on abdominal segment V as microsetae, and less differentiated sensory seta p3 on abdominal segment V. Bisexual.

##### Remarks.

The presence of pseudocelli on thoracic segment I easily distinguishes *Paratullbergia changfengensis* sp. n. from other congeners. It is similar to *Paratullbergia trivandrana* Prabhoo, 1971 from India in the presence of two pairs of pseudocelli on each of thoracic segments II and III, but differs in the presence of pseudocelli on thoracic segment I (absent in *Paratullbergia trivandrana*) and shape of sensory seta p3 on abdominal segment V (setiform vs. flame-like). The nine existing species of the genus *Paratullbergia* can be distinguished by the following key.

### Key to the species of genus *Paratullbergia* Womersley, 1930 modified from Dunger and Schlitt 2011.

**Table:** 

1	Th I–III without pso	***Paratullbergia concolor* Womersley, 1930** (UK)
–	Th I–III with pso	**2**
2	Th II and III with 2+2 pso	**3**
–	Th II and III with 1+1 pso	**4**
3	Pso present on Th I, sensory seta p3 on Abd V setiform	***Paratullbergia changfengensis* sp. n**.(China)
–	Pso absent on Th I, sensory seta p3 on Abd V flame-like	***Paratullbergia trivandrana* Prabhoo, 1971** (India)
4	Abd VI between the crescentic ridges and Asp with 1+1 rounded tubercles	***Paratullbergia callipygos* (Börner, 1902)** (Holarctic)
–	Abd VI without additional tubercles	**5**
5	PAO with less than 25 vesicles	**6**
–	PAO with more than 35 vesicles	**7**
6	Abd II and III with pso, PAO with 22–24 vesicles	***Paratullbergia indica* Salmon, 1965** (India)
–	Abd II and III without pso, PAO with 16–18 vesicles	***Paratullbergia salmon* Prabhoo, 1971** (India)
7	Ant IV with 4 thickened sensilla; on Abd I a4 as microseta, Abd II and III without m3	***Paratullbergia macdougalli* Bagnall, 1936** (Palaearctic)
–	Ant IV with 5 thickened sensilla; on Abd I a4 as macroseta; Abd II and III with m3	**8**
8	PAO with 35–40 vesicles	***Paratullbergia caroli* Luciáňez, Ruiz & simón, 1991** (Spain)
–	PAO with 64–68 vesicles	***Paratullbergia brevispina* Skarżyński & Pomorski, 1999** (Turkey)

## Supplementary Material

XML Treatment for Paratullbergia changfengensis

## References

[B1] Bellinger PF Christiansen KA Janssens F (1996–2015) Checklist of the Collembola of the World. http://www.collembola.org [accessed 28 April 2015]

[B2] Bu Y Potapov MB (2013) A new species and new records of Pachytullbergiidae and Tullbergiidae (Collembola: Onychiuroidea) from littoral of China, with notes on the variations of postantennal organ. Zootaxa 3669(2): 139–146. doi: 10.11646/zootaxa.3669.2.4 26312328 10.11646/zootaxa.3669.2.4

[B3] Dunger W Schlitt B (2011) Synopses on Palaearctic Collembola: Tullbergiidae. Soil Organisms 83(1): 1–168.

[B4] Gao Y (2007) Studies on the Systematics of Collembola and Soil Zooecology. PhD Dissertation, Shanghai Institutes for Biological Sciences, Chinese Academy of Sciences, Shanghai, 99 pp.

[B5] Prabhoo NR (1971) Soil and litter Collembola of south India 1. Arthropleona. Oriental Insects 5(1): 1–46. doi: 10.1080/00305316.1971.10433988

[B6] Rusek J (1967) Beitrag zur Kenntnis der Collembola (Apterygota) Chinas. Acta Entomologica Bohemoslovaca 64: 184–194.

[B7] Rusek J (1991) New Holarctic and Palearctic taxa of Tullbergiinae (Collembola). Acta Societatis Zoologicae Bohemoslovacae 55(1/2): 65–75.

[B8] Tamura H Zhao L (1996) Two species of the subfamily Tullbergiinae in Xishuangbanna, southwest China (Collembola; Onychiuridae). Japanese Journal of Entomology 64(4): 790–794.

